# Functional networks and structural connectivity of visuospatial and visuoperceptual working memory

**DOI:** 10.3389/fnhum.2015.00340

**Published:** 2015-06-11

**Authors:** Roser Sala-Llonch, Eva M. Palacios, Carme Junqué, Núria Bargalló, Pere Vendrell

**Affiliations:** ^1^Department of Psychiatry and Clinical Psychobiology, University of BarcelonaBarcelona, Spain; ^2^Institute of Biomedical Research August Pi i SunyerBarcelona, Spain; ^3^Centre de Diagnòstic per la Imatge Clínic, Hospital Clínic de BarcelonaBarcelona, Spain

**Keywords:** fMRI, DTI, tractography, fusiform, facial working memory

## Abstract

Neural correlates of working memory (WM) in healthy subjects have been extensively investigated using functional MRI (fMRI). However it still remains unclear how cortical areas forming part of functional WM networks are also connected by white matter fiber bundles, and whether DTI measures, used as indices of microstructural properties and directionality of these connections, can predict individual differences in task performance. fMRI data were obtained from 23 healthy young subjects while performing one visuospatial (square location) and one visuoperceptual (face identification) 2-back task. Diffusion tensor imaging (DTI) data were also acquired. We used independent component analysis (ICA) of fMRI data to identify the main functional networks involved in WM tasks. Voxel-wise DTI analyses were performed to find correlations between structural white matter and task performance measures, and probabilistic tracking of DTI data was used to identify the white matter bundles connecting the nodes of the functional networks. We found that functional recruitment of the fusiform and the inferior frontal cortex was specific for the visuoperceptual working memory task, while there was a high overlap in brain activity maps in parietal and middle frontal areas for both tasks. Axial diffusivity and fractional anisotropy, of the tracts connecting the fusiform with the inferior frontal areas correlated with processing speed in the visuoperceptual working memory task. Although our findings need to be considered as exploratory, we conclude that both tasks share a highly-overlapping pattern of activity in areas of frontal and parietal lobes with the only differences in activation between tasks located in the fusiform and inferior frontal regions for the visuoperceptual task. Moreover, we have found that the DTI measures are predictive of the processing speed.

## 1. Introduction

Working Memory (WM) refers to the capacity to maintain, manipulate and store information during short periods of time. It involves a set of brain structures and processes to organize and integrate different kinds of information (Baddeley, 1986). Magnetic Resonance Imaging (MRI) has been used to investigate brain networks involved in WM both at the functional and structural levels, by means of functional MRI (fMRI) and Diffusion Tensor Imaging (DTI), respectively.

The use of fMRI to measure blood-oxygen-level-dependent (BOLD) signal during working memory tasks has evidenced consistent activation of frontal and parietal cortical regions regardless of the stimulus modality (D'Esposito et al., 1998; Wager and Smith, 2003; Owen et al., 2005). These regions include the bilateral posterior parietal cortex, the bilateral premotor cortex, the dorsal cingulate/medial premotor cortex, the frontal pole, and the bilateral dorsolateral-midventrolateral prefrontal cortex. However, some studies have reported domain-specific activation within brain structures (Fuster, 1997; Rottschy et al., 2012). Working memory tasks activate the lateral prefrontal region and, concomitantly, a region of the posterior cortex that varies according to the sensory modality: visual stimuli activate in general the inferior temporal and parastriate cortex, auditory input activates the superior temporal cortex, and spatial stimuli produce activity in the posterior parietal cortex (Fuster and Bressler, 2012). As regards the involvement of visual regions, it is possible to differentiate between the dorsal and ventral processing streams, both originating in the striate cortex. The ventral stream passes through the occipitotemporal cortex to its anterior temporal target and to the ventrolateral prefrontal cortex. And the dorsal stream goes from the occipitoparietal cortex to the posterior half of the inferior parietal lobule and the dorsolateral prefrontal cortex. Recently, it has been concluded that the dorsal stream gives rise to three distinct major pathways namely the parieto-prefrontal, parieto-premotor and parieto-medial temporal pathways which support spatial working memory, visually guided action, and spatial navigation respectively (Kravitz et al., 2011). In addition, studies on WM for spatial locations have reported increased activity in the right inferior parietal lobule and left insula (Passaro et al., 2013). Therefore, we suggest that the simultaneous investigation of various working memory paradigms using different kinds of stimuli may help to identify stimulus-specific regions of activity.

In addition, DTI allows the measurement of microstructural properties of brain white matter. DTI indices, such as Fractional Anisotropy (FA), Radial Diffusivity (RD) and Axial Diffusivity (AD) relate to white matter integrity, since they are thought to reflect the degree of myelination, axonal membrane thickness and axon diameter (Beaulieu, 2002; Song et al., 2002). DTI probabilistic tractography is a complex but an effective tool that can reconstruct *in vivo* the trajectories of white matter fasciculi connecting different cortical areas (Behrens et al., 2003; Catani and Thiebaut de Schotten, 2008). Using DTI, it has been found that FA values correlate positively with working memory performance of the subjects (Olesen et al., 2003; Klingberg, 2006) as well as with task-related BOLD activity during WM (Burzynska et al., 2011). In this regard, the superior longitudinal fasciculus (SLF) has been identified as the main tract involved in the WM network. The relationship between SLF integrity and working memory has also been studied in pathologies such as multiple sclerosis, schizophrenia, and traumatic brain injury (Audoin et al., 2007; Karlsgodt et al., 2008; Palacios et al., 2011). Finally, in a more general context, it has been reported that the structural integrity of major white matter tracts, including the callosal genu and splenium, the cingulum, optic radiations and the superior longitudinal fasciculus, correlates with the performance intelligence quotient, and that this relationship is mediated by genetics (Chiang et al., 2012). In children, high FA values have been linked to improved response inhibition, enhanced working memory, and faster reaction times (Madsen et al., 2011; Vestergaard et al., 2011). In adults, high FA values in parietal and frontal white matter were associated with faster performance on a lexical decision task (Gold et al., 2007) and faster reaction times for tasks involving visuospatial attention (Tuch et al., 2005).

Although many studies have described the functional and structural properties of working memory networks, in the current study we add a novel approach to the field by using available advanced neuroimaging techniques. We aim to define functional networks with ICA and to study structural connectivity with DTI of two different tasks involving different brain networks guided by the stimuli. Our main goals are: (1) to identify the differences in the task-activated networks for spatial and facial working memory, and (2) to describe the structural connectivity of these networks measured with DTI indices.

Whereas brain functional connectivity can be studied as the temporal correlation between spatially remote neuropsychological events during the performance of a cognitive task (Biswal et al., 1995), structural connectivity refers to the presence of fiber tracts directly connecting regions (Rykhlevskaia et al., 2008). Our aim was to combine data-driven fMRI analysis with probabilistic tractography and DTI maps to determine the functionality and connectivity between brain regions involved in visual working memory tasks and their relationship with cognitive performance. For this purpose, we used independent component analysis of fMRI data and a whole-brain analysis of DTI-derived maps. In our two WM tasks, different stimuli were used to determine the possible domain-specificity of the neural basis of working memory: one n-back task involving visuospatial processing (squares), and a second n-back task using visuoperceptual stimuli (faces). In summary, by doing exploratory analyses, we aimed to test the hypothesis that the two tasks would activate different functional networks. Moreover, we wanted to explore the structural connectivity of these networks under the hypothesis that microstructural properties of the white matter connections would be predictive of task performance.

## 2. Materials and methods

### 2.1. Subjects and acquisition

Twenty-three healthy young subjects (mean age: 28.26, SD: 6.76, 12 males, 11 females) with no history of psychiatric or neurological pathologies were included in the study. The study was approved by the research ethics committee of the University of Barcelona and participants gave written informed consent. Subjects were scanned on a 3T MRI scanner (Magnetom Trio Tim, Siemens Medical Systems, Germany) during the performance of the working memory tasks, using a single shot gradient-echo EPI sequence (TR = 2000 ms; TE = 16 ms; FOV = 220 x 220 mm^2^; voxel size = 1.7 x 1.7 x 3.0 mm; flip angle = 90 degrees). High resolution T1-weighted images were acquired with the MPRAGE 3D protocol (TR = 2300 ms; TE = 3 ms; TI = 900 ms; FOV = 244 x 244 mm^2^; 1 mm isotropic voxel) and diffusion-weighted images were sensitized in 30 non-collinear directions with a *b*-value = 1000 s/mm^2^, using an echo-planar (EPI) sequence (TR = 9300 ms, TE = 94 ms, slice thickness = 2 mm, voxel size = 2 x 2 x 2 mm, FOV = 240 x 240 mm^2^, no gap).

During fMRI acquisition, the task was projected in a big screen outside the scanner, and shown to the subject using a mirror system placed in front of subject's eyes. The subject was provided with a response button, also synchronized with the stimuli presentation, and responses were recorded in a computer outside the scanner. The size of the projected image was 40 x 50 cm, and it was placed at a distance of 2 m from the subjects head. The vertical distance between subjects eyes and the center of the image was 5 cm, so the visual angle was nearly 0. Tasks were presented and synchronized with functional acquisition using the Presentation® software (NeuroBehavioral Systems, NBS).

The two cognitive tasks presented during the MRI scanning consisted on 2-back WM paradigms. In each of them, a sequence of stimuli was presented on the screen and the subject was asked to indicate whether the stimulus was identical to the one shown 2 trials before. For the assessment of visuoperceptual working memory, we used facial images from an available database (Minear and Park, 2004). For the visuospatial working memory task, the stimuli were color squares located in different positions on a black screen. In both tasks, a 0-back task was used as a control condition, and subjects were asked to indicate whether the current trial matched a specific stimulus. For the facial task, in the control blocks, subjects were asked to press the button when the person that appeared in the screen was wearing glasses. For the spatial control task, subjects were asked to indicate if the color square shown was placed in the middle of the screen. Stimuli sizes were 55 and 74% of the screen width for the squares and faces respectively.

During the scanning session, 12 subjects underwent first the facial 2-back task and then the spatial 2-back task, while the remaining subjects performed the tasks in the opposite order.

Within each task, the sequence of stimuli was presented using a block-design paradigm, where 2-back blocks were alternated with 0-back blocks. Within each block, a total number of 14 stimuli were presented. Each image appeared on the screen for 1 s, with an interstimulus interval (black screen) of 1 s. An instruction screen was presented at the beginning of each block for 2 s. There were 16 blocks, alternating between 0-back and 2-back conditions, presented in the course of the 8-min experiment for each task. Individual responses were collected, and performance scores were computed using the d-prime measure [Z(hits rate) − Z(false alarm rate)](Macmillan and Creelman, 1991). In addition, mean reaction times (RT) separately for each task and condition were collected, where RT was measured as the time between the stimulus onset and the subject's response.

### 2.2. Analysis of fMRI data

Functional MRI data were analyzed using Multivariate Exploratory Linear Optimized Decomposition into Independent Components (MELODIC) (Beckmann and Smith, 2004), as implemented in FSL (http://www.fmrib.ox.ac.uk/fsl). Before ICA decomposition, the preprocessing of fMRI data included motion correction using MCFLIRT (Jenkinson and Smith, 2001), removal of non-brain regions with BET (Smith, 2002), spatial smoothing using a Gaussian kernel of FWHM 5 mm, grand-mean intensity normalization and high-pass temporal filtering (using FWHM = 160 s).

Then, three different ICA decompositions were carried out. First, fMRI data from facial and spatial tasks were analyzed separately, and a third analysis was performed with data from the two tasks. In each case, ICA decomposed functional data into a set of spatio-temporal Independent Components (ICs). Each IC was composed by a spatial map, an associated time-course and a subjects mode vector, indicating the strength of the component for each subject.

For each task-separated analysis, we identified the components related with the 2-back>0-back contrast and we selected the IC with the best fit to the task time-series. This procedure allowed the identification of the main functional network associated with spatial WM processing (spatial IC), and facial WM processing (facial IC). In addition, with the ICA analysis performed with data from the two tasks, we could identify components of higher activity in one on the two tasks with respect to the other.

### 2.3. Definition of ROIs from functional data

We used the maps obtained in the two separate ICA analyses to create a set of ROIs that were common between the two tasks as well as one ROI that was specific for the visuoperceptual task. For this, we proceeded as follows: For each task, we selected the component having the best temporal fit with the task timeseries and we thresholded its spatial map (*Z*>2.3) to obtain the clusters of task-related activity (summarized in Table 1). Peak voxels of these regions were identified with an atlas-based region using the Harvard-Oxford atlas available in FSL. For the regions activated commonly in both tasks, we created the corresponding tractography-ROI by calculating the overlap between the two ICA-clusters and the atlas-derived mask. For the fusiform ROI, we calculated the overlap between the facial-IC and the fusiform mask from the atlas.

**Table 1:** **Definition and localization of the ROIs from functional data**.

**ROI id**	**Visuospatial WM Task (*Spatial*)**	**visuoperceptual WM task** **(*Facial*)**
Fus ROI	–	L: coord.: (−10, −82, −4), *Z* = 3.59, size = 2240 mm^3^ ^a^
	–	R: coord.: (34, −62, −8), *Z* = 2.85, size = 128 mm^3^
IF ROI	L: coord.: (−42, 30, 28), *Z* = 4.70, size = 1536 mm^3^	L: coord.: (−42, 26, 28), *Z* = 4.48, size = 7104 mm^3^
	R: coord.: (34, 38, 28), *Z* = 3.15, size = 384 mm^3^	R: coord.: (34, 54, 12), *Z* = 4.21, ^b^
Ins ROI	L: coord.: (−30, 22, 0), *Z* = 2.75, size = 64 mm^3^	L: coord.: (−30, 22, 4), *Z* = 2.98, size = 128 mm^3^
	R: coord.: (30, 22, 0), *Z* = 3.76, size = 832 mm^3^	R: coord.: (30, 22, 0), *Z* = 5.52, size = 5696 mm^3^
MF ROI	L: coord.: (−30, 2, 56), *Z* = 7.39, size = 8896 mm^3^	L: coord.: (−34, 6, 56), *Z* = 4.59, size = 3328 mm^3^
	R: coord.: (22, 2, 52), *Z* = 6.49, size = 7616 mm^3^	R: coord.: (34, 10, 56), *Z* = 6.45, size = 43136 mm^3^, ^b^
Par ROI	L: coord.: (−14, 66, 60), *Z* = 9.16, size = 68544 mm^3^, ^c^	L: coord.: (−25, −58, 52), *Z* = 5.56, size = 46848 mm^3^, ^d^
	R: coord.: (10, 62, 64), *Z* = 11.5, ^c^	R: coord.: (38, −46, 56), *Z* = 6.38, ^d^
Temp ROI	L: –	L: –
	R: coord.: (50, −58, 4), *Z* = 3.7, size = 576 mm^3^	R: MNI coord.: (54, −42, 0), *Z* = 3.49, size = 1344 mm^3^

*L, Left hemisphere; R, Right hemisphere; Fus, fusiform; IF, Inferior Frontal; Ins, Insula; MF, Middle Frontal; Par, Parietal; Temp, Temporal; coord, coordinates (in MNI space)*.

*Z-values indicate the Z score of the IC at the peak maxima reported by the MNI coordinates*.

a *Secondary maximum at MNI coordinates: (−30, 14, 15), with Z = 3.20*.

b *Cluster size comprising the right-IF and right-MF ROIs*.

c *Cluster size comprising the left-Par and right-Par ROIs*.

d *Cluster size comprising the left-Par and right-Par ROIs*.

Overall, we created 6 ROIs in the right hemisphere and 5 ROIs in the left hemisphere (Table 1): the Fusiform ROI (*Fus ROI*, bilateral), the Inferior Frontal ROI (*IF ROI*, bilateral), the Insular ROI (*Ins ROI*, bilateral), the Middle Frontal ROI (*MF ROI*, bilateral), the Parietal ROI (*Par ROI*, bilateral) and the Temporal ROI (*Temp ROI*, right hemisphere). The Fus ROI was specific for the facial task, whereas the other ROIs were calculated as the overlap between the activity maps of the two tasks.

It should be noted from Table 1 that in some cases, the clusters obtained from the thresholded ICA maps expanded to more than one functional region, despite having two clear peaks of activation. In this sense, we observed that the activation in right-IF and right-MF defined a single cluster for the visuoperceptual task, and that left-Par and right-Par ROIs activity peaks were included in the same big cluster for both the visuospatial and the visuoperceptual tasks. In these cases, for tractography purposes, the final ROIs were defined by masking the cluster with the two corresponding atlas-derived regions, separately.

### 2.4. Preprocessing of diffusion MRI data

Diffusion MRI Images were analyzed using FDT (FMRIB's Diffusion Toolbox), from FSL (Behrens et al., 2007). Firstly, data were corrected for distortions caused by the eddy currents in the gradient coils and for simple head motion, using the B0 non-diffusion data as a reference volume. Then, Fractional Anisotropy (FA) maps from each subject were obtained using a diffusion tensor image (DTI) model fit. With DTI, we also obtained individual maps for axial diffusivity (AD) and radial diffusivity (RD). Maps were registered and projected to a common skeleton map using the TBSS algorithm (Smith et al., 2006). Further, we performed voxel-wise statistics between subjects using these maps.

In addition, a probabilistic tractography algorithm was applied to the diffusion images (Behrens et al., 2007). Tractography was used to estimate the connectivity between pairs of ROIs (seed ROI and end ROI) individually for each subject, resulting in maps that indicated the probability of each voxel to have a tract connecting both ROIs. The entire probabilistic tracking procedure was carried out in each subjects anatomical space. Using the probabilistic tractography algorithm, we obtained individual maps for each pair of ROIs, where each voxel value indicated the probability of having fibers connecting the two regions. These maps were thresholded (at 2% of their maximum) in order to remove very-low probability fiber paths. Individual FA scores inside each pathway were calculated as the mean FA of all voxels in the tract and they were then used to quantify and compare the integrity of the identified paths. Tract-averaged scores for axial diffusivity (AD) and radial diffusivity (RD) were also obtained for the main tracts of interest.

### 2.5. Statistical analyses

Statistical comparison between activation maps were performed using the subjects' vector from the whole-group (facial and spatial) ICA decomposition. These vectors had an score for each subject and task that indicates the activation of a given component. They were introduced into PASW (Statistical Package for Social Sciences, Chicago, II, USA) and we evaluated differences between tasks using paired *t*-test analysis of these values.

Correlations between whole-brain DTI indices and performance scores were evaluated using a General Lineal Model (GLM) in FSL. The resulting maps were corrected for multiple comparisons using randomize from FSL (Nichols and Holmes, 2002). This method is a permutation method that is used for inference on statistic maps when the null distribution is not known. In our analyses, the number of permutations was set to 5000.

In addition, scores of mean FA, AD, and RD were obtained within the tracts of interest derived from the DTI-tractography analysis. We used Pearson's correlation in PASW to investigate correlations between the measures of FA, AD, and RD within the tracts and cognitive performance.

## 3. Results

### 3.1. Task performance

All subjects showed high task performance measured by d prime measure. A summary of behavioral measures for each task and condition is shown in Table 2. We found significant differences between 0-back and 2-back tasks for both RT and d prime measures and for both spatial and facial tasks. In general, subjects were slower in respond and performed worse in the 2-back conditions compared with 0-back conditions (*p* < 0.001 in paired *T*-Tests).

**Table 2:** **Behavioral results of the two working memory tasks for both conditions**.

**fMRI Task**	**d prime mean(SD)**	**Response time mean(SD)**
Spatial 0-back	4.03 (0.31)	0.416 (0.08) s
Spatial 2-Back	3.49 (0.40)	0.47 (0.09) s
Facial 0-back	4.08 (0.28)	0.495 (0.07) s
Facial 2-Back	3.33 (0.65)	0.546 (0.09) s

### 3.2. Identification of functional networks involved in spatial and facial working memory

**Figure 1:**
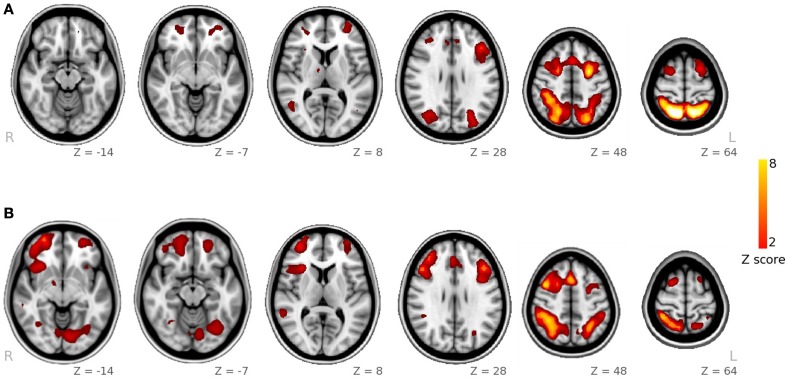
**Spatial maps obtained from ICA decomposition of functional data**. **(A)** Main component for the spatial WM task; **(B)** main component for the facial WM task.

Using Tensor-ICA (T-ICA) for the whole group of 23 subjects and separately for each task, we identified the functional pattern for spatial and facial working memory processes (named spatial IC and facial IC, Figure 1). Spatial IC covered regions of the frontal and parietal cortices, with the main foci of activation in middle frontal and parietal regions. The Facial IC, shared the middle frontal and parietal regions of activity, but it showed additional activity in areas of the fusiform and in a region of the inferior frontal. Additionally, we identified foci of activation in the Insula and in a region of the temporal cortex. As explained in the methods section, the coordinates of all the foci as well as the size of the corresponding clusters are reported in Table 1.

With the ICA analysis of the facial and the spatial task together, we identified a component, associated with the 2-Back>0-back contrast, with higher activity during facial WM than during spatial WM (*p* = 0.01 in the paired-samples *t*-test analysis). Its spatial map included the left inferior frontal frontal gyrus, a small region in the right middle frontal gyrus, part of the paracingulate gyrus, and a cluster located in the occipital pole, occipital fusiform, the lingual and fusiform gyrii and the inferior division of the lateral occipital cortex.

### 3.3. Whole-brain DTI analysis

**Figure 2:**
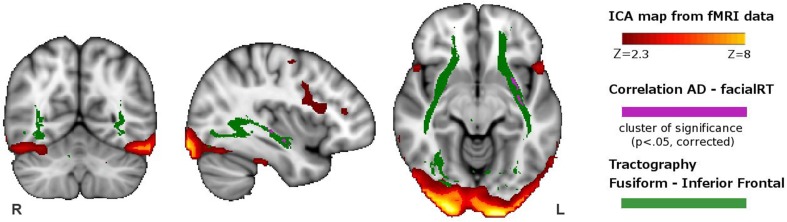
**Overlapping of the functional and structural results**. In red-yellow, we show the spatial map of the ICA-component having greater activity during visuoperceptual (facial) compared with visuospatial WM. In violet, we depict the regions showing significant correlation between axial diffusivity and reaction time from the whole-brain DTI analysis. In green, we indicate the tract connecting the fusiform with the inferior frontal ROI, obtained with DTI probabilistic tractography.

In the voxel-wise analysis of maps derived from DTI analysis, we identified a cluster where AD correlated negatively with RT of the facial WM task (*p*< 0.05, corrected). This cluster was located in the left inferior fronto-occipital fasciculus and left uncinate fasciculus (Figure 2).

### 3.4. DTI tractography

We used the spatial pattern of the structural connection between the fusiform and the inferior fronta ROIs in orderl to demonstrate that it overlapped the region where AD correlated with response time of the facial WM task (Figure 2). This connection included the inferior fronto-occipital fasciculus, inferior longitudinal fasciculus, part of the uncinate fasciculus and part of the anterior thalamic radiation.

**Figure 3:**
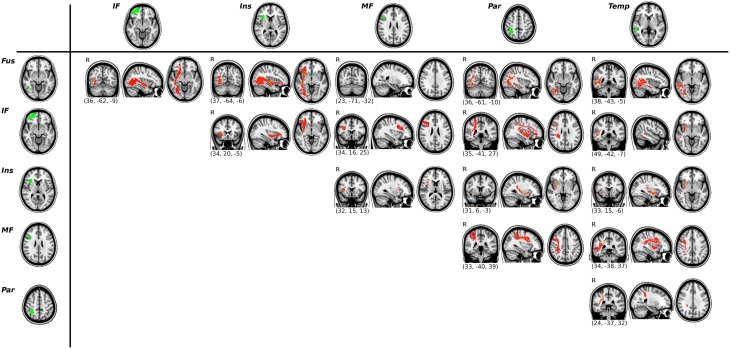
**Results of the ROI-to-ROI tractography for the right hemisphere**. Functional ROIs are shown in green (left column and upper row) and their corresponding white matter connections are displayed in red. All tracts are thresholded at their 2% and averaged across all subjects.

Apart from this connection, we used probabilistic tractography to obtain the main tracts involved in the functional tasks. In total, 15 ROI-to-ROI pathways were identified in each hemisphere (See Figure 3 for the tracts of the right hemisphere, and Supplementary Figure 1 for the tracts of the left hemisphere).

### 3.5. Correlation between DTI-tractography measures and task performance

We conducted exploratory correlational analyses to evaluate associations between task measures and DTI parameters of the tracts identified previously (i.e., all the ROI-to-ROI paths shown in Figure 3). The mean FA values of the tracts connecting the Fus ROI and the IF ROI correlated with RT scores during the visuoperceptual (facial) WM task (*r* = −0.639, *p* = 0.001 for the right hemisphere, *r* = −0.464, *p* = 0.030 for the left hemisphere). Axial Diffusivity (AD) of the same pathway (Fus to IF ROIs) also correlated with RT scores of the visuoperceptual (facial) WM task (*r* = −0.449, *p* = 0.036 in the right hemisphere and *r* = −0.603, *p* = 0.003 in the left hemisphere).

In addition, we observed a significant correlation between d prime measures of the visuospatial WM task and RD indices in the tracts connecting the Ins ROI and the MF ROI of the left hemisphere (*r* = −0.447, *p* = 0.037).

All the previous correlations are reported at the uncorrected level of significance. Using Bonferroni correction for multiple comparisons, we found that only the correlation between mean FA in the right Fus-IF tract and RT in the visuoperceptual WM task remained significant. Given this, it should be mentioned that the correlations between task measures and DTI parameters should be interpreted with caution. Finally, in order to ensure that the correlations were not driven by outliers, we created scatter plots of all the significant results (see Supplementary Figure 2).

## 4. Discussion

By using Independent Component Analysis of fMRI data and DTI, we studied patterns of brain activity and structural connectivity related with two working memory tasks: a visuospatial task involving working memory for spatial locations and a visuoperceptual task with facial stimuli. We observed that both tasks share a highly-overlapping pattern of activity in areas of frontal and parietal lobes and that the only differences in activation between tasks were located in the fusiform and inferior frontal regions for the visuoperceptual task. With DTI, we first performed a whole-brain analysis and we found a region in the left inferior fronto-occipital fasciculus and the left uncinate where AD correlated with the response time in the visuoperceptual WM task. Furthermore, we performed a second analysis, which was a ROI-to-ROI tractography in order to confirm the results obtained with whole-brain DTI analyses. We found that FA scores within the tract connecting the fusiform and the inferior frontal ROIs also correlated with reaction time in the visuoperceptual task.

The shared pattern of fMRI activity from both tasks coincides with the core working memory network. We observed bilateral activation of the superior parietal lobule, frontal pole, dorsolateral-midventrolateral prefrontal cortex, and the anterior insula. These activated regions coincide with those described in several meta-analyses of the n-back task (Fuster, 1997; Owen et al., 2005; Rottschy et al., 2012). We also found brain activity involving the ventral and lateral intraparietal region and the mid-temporal area, which receives strong input from visual processing regions. For the facial working memory task, in addition to the fronto-parietal and mid-temporal pattern of activity, we observed increased activity in the fusiform region and in the inferior parietal gyrus. This is in agreement with previous works showing the involvement of the fusiform area in face processing, including the detection and discrimination of faces (McCarthy et al., 1997; Haxby et al., 2001; Kanwisher, 2010). It should be noted that the design of the task could not differentiate the specific processes of face identification and facial memory, because the control task did not specifically require face identification, and therefore, the involvement of the fusiform could also be explained by differences in perceptual processing. In this regard, some authors have reported that the fusiform is activated during face-memory processes (Simó et al., 2015), but there are also evidences of its implication in face perception and face processing (Grill-Spector and Malach, 2004; Rieck et al., 2015). In general, it seems difficult to differentiate whether the fusiform activity appears as a response of the processing of faces or as part of the facial memory process. In our case, the control stimuli were subjects wearing glasses. That is, it required the identification of an object (i.e., glasses), but not the processing of a face. Given this, we suggest that the fusiform may be already involved in the processing face before its retention.

In the spatial task, the shape processing, which could be considered a visuoperceptual process, was controlled by the task design, as the control task involved the same shape and color figure as the target task; therefore, we did not identify the participation of any region of the ventral stream. As expected due to the nature of the task, the superior parietal region of the dorsal stream was involved (Rottschy et al., 2012).

As regards the DTI analysis, we performed two kinds of analyses, which were a whole-brain analysis of DTI-derived maps and a ROI-based analysis of the tracts depicted from the functional task-activations.

In the whole-brain DTI analysis, we found a cluster in the left inferior fronto-occipital fasciculus and the uncinate where AD correlated with RT for the facial WM task. In addition, by doing probabilistic tractography, we identified the main tracts involved in the functional networks and we found that mean FA and mean AD scores within the pathway connecting the fusiform and the inferior frontal ROIs also correlated with facial RT. In all cases, subjects with higher AD or FA scores had faster reaction times. Therefore, exploratory correlational analyses of the tracts support and extend the results obtained with the whole-brain DTI analysis.

Previous studies have reported that faster reaction time is associated with higher FA values measured with DTI. In adult subjects, Gold et al. (2007) found that the speed of visual word recognition correlated with tract integrity, measured with FA in parietal regions; and Tuch et al. (2005) found that reaction time of visuospatial learning also correlated with FA values in the parietal region.

In our results, the whole-brain analysis showed a negative correlation between AD and response time in the left hemisphere, which differs from other studies showing right hemispheric preference in the correlation between DTI and performance in facial recognition (Tavor et al., 2014). It has been suggested that AD is a more putative measure of axonal integrity, providing information about the status of the axons (Di Paola et al., 2010) and that it could be more directly related with the capacity of the brain white matter to conduct information between different brain regions (Lazar et al., 2014). Furthermore, studies using animal models with inflammatory and demyelinating lesions have concluded that AD reflects axonal transport properties (DeBoy et al., 2007; Budde et al., 2009). Although we could not find a specific explanation for the fact that the correlations with AD were found mainly in the left hemisphere, we suggest that, considering that AD is more directly associated with processing speed, its correlations would be found more generally in areas reflecting bilateral antero-posterior communication. In conclusion, our results suggest that AD could be measuring axonal properties that are directly related to the capacity to conduct information between brain regions (Lazar et al., 2014), whereas RD is related to myelination and measures properties of brain maturation with a more direct impact on task performance.

As regards the rest of results of tractography analyses, first evidencied that all ROI pairs resulted in tractography maps that included several tracts, and that were in accordance with reported structural brain connections (Catani et al., 2002). We found a correlation in the tract connecting the insula and the middle frontal ROIs in the left hemisphere. That is, the RD of this tract correlated with the d prime score in the visuospatial task. Regions in the left hemisphere, ans specially the left insula have been previously associated with the maintenance of information for object location in fMRI studies (Passaro et al., 2013). In this case, as opposite to the facial task, the correlation was found only with RD index. It has been suggested that RD is a measure of the degree of myelination and reflects processes of brain maturation or degeneration (Song et al., 2002), and it has been related to task performance in several studies (Tamnes et al., 2010; Østby et al., 2011; Tavor et al., 2014).

It should be noted that apart from this correlation, we did not find any strong evidence of a structure-performance relationship in the spatial WM task. We suggest several explanations for this lack of correlation. One possibility is that task-performance may be the result of more complex network interactions between structure and function in both positive and negative task-networks, as suggested in other reports (Burzynska et al., 2013). In addition, this lack of relatiosnhips may also be attributable to divergences between structure and function, and the fact that the tracts identified in the structural network are shared by different functional networks (Park and Friston, 2013). In this case, some of the fasciculi, like the SLF, would not be specific for the spatial WM task.

In summary, we have characterized the functional and structural networks of working memory for spatial and facial stimuli. We concluded that these networks share the fronto-parietal connections, and that facial working memory involves additional recruitment of fusiform and inferior frontal regions. Furthermore, the DTI results highlighted the relationship between microstructural properties of the inferior fronto-occipital fasciculus and reaction time in the visuoperceptual working memory.

The present study underlines the importance of analyzing both the function and the structure of brain networks.

This study has several limitations. First, the number of subjects is relatively small. Other limitations are related to the design of the fMRI study itself, for example, the lack of control for eye movements, which could affect the differences between activity patterns. In this regard, it has been reported that the activity associated with eye movements is located in the precentral sulcus, close to its junction with the superior frontal sulcus (Grosbras et al., 2005; Vernet et al., 2014), which are different from the areas reported in our study. Therefore, we conclude that our results are not likely to be biased by differences in eye movements between tasks. In addition, another limitation is that the control conditions used for spatial and facial WM are not exactly equivalent, so it is difficult to discern if the activity in the fusiform cortex is related with facial processing or with facial memory. We also acknowledge that fMRI data were acquired at a short TR, which is not the optimal for GE EPI BOLD measurements, which should be considered in replications of the study.

To conclude, we recognize that further experiments need to be done to validate our results. For example, it would be very useful to design an experiment with comparable control conditions in each task, able to isolate the facial processing component from the facial memory component. In addition, we suggest that the inclusion of fixation periods in the tasks would facilitate controlling for differences in eye-movements. Finally, another possible improvement to the task would be to mix the visuoperceptual and the visuospatial conditions in a single fMRI-block design, with alternating blocks of each condition. This would imply a longer fMRI acquisition, but it would facilitate the data analysis and interpretation.

### Conflict of interest statement

The authors declare that the research was conducted in the absence of any commercial or financial relationships that could be construed as a potential conflict of interest.
