# Supplementary material for: Functional networks and structural connectivity of visuospatial and visuoperceptual working memory
Source: Front Hum Neurosci. 2015 Jun 11;9:340. doi: 10.3389/fnhum.2015.00340 (PMC4463024; doi:10.3389/fnhum.2015.00340)
Supplement: Supplementary file 1 [file Presentation1.PDF]

FUNCTIONAL NETWORKS AND STRUCTURAL CONNECTIVITY OF VISUOSPATIAL AND VISUOPERCEPTUAL WORKING MEMORY

Roser Sala-Llonch, Eva M. Palacios, Carme Junqué, Núria Bargalló and Pere Vendrell.

SUPPLEMENTARY MATERIAL.

Supplementary Figure 1:

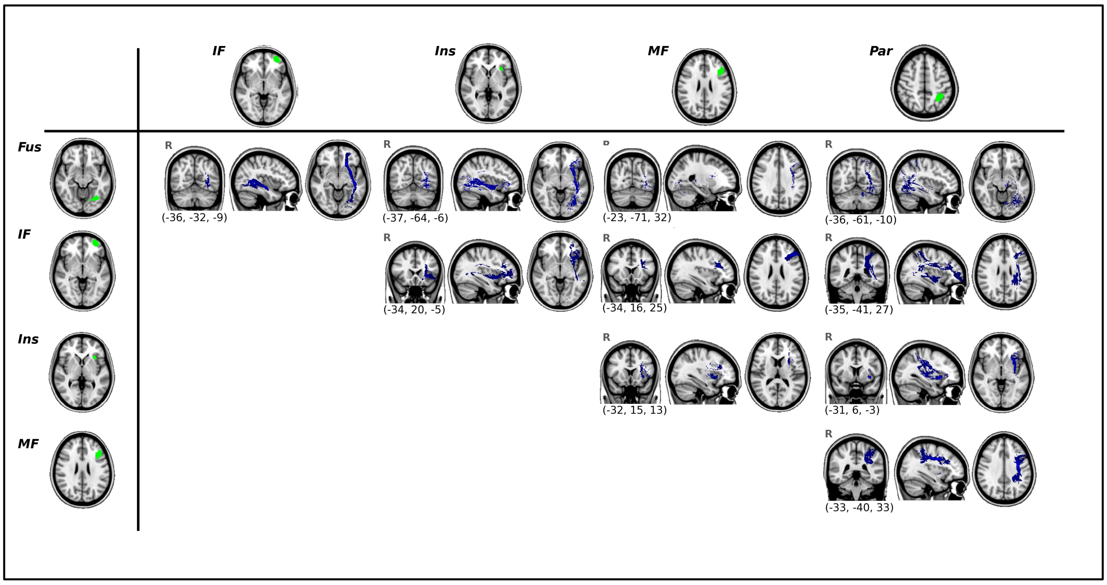

Supplementary Figure 1: Results of the ROI-to-ROI tractography for the left hemisphere. In green: masks of the 5 ROIS (see table 1 for description). In blue: probabilistic tractography maps, averaged across all subjects. MNI coordinates of each image are indicated in mm.

Fus: Fusiform ROI; *IF:* Inferior Frontal ROI; *Ins:* Insula ROI; *MF:* Middle Frontal ROI; *Par:* Parietal ROI.

Supplementary Figure 2:

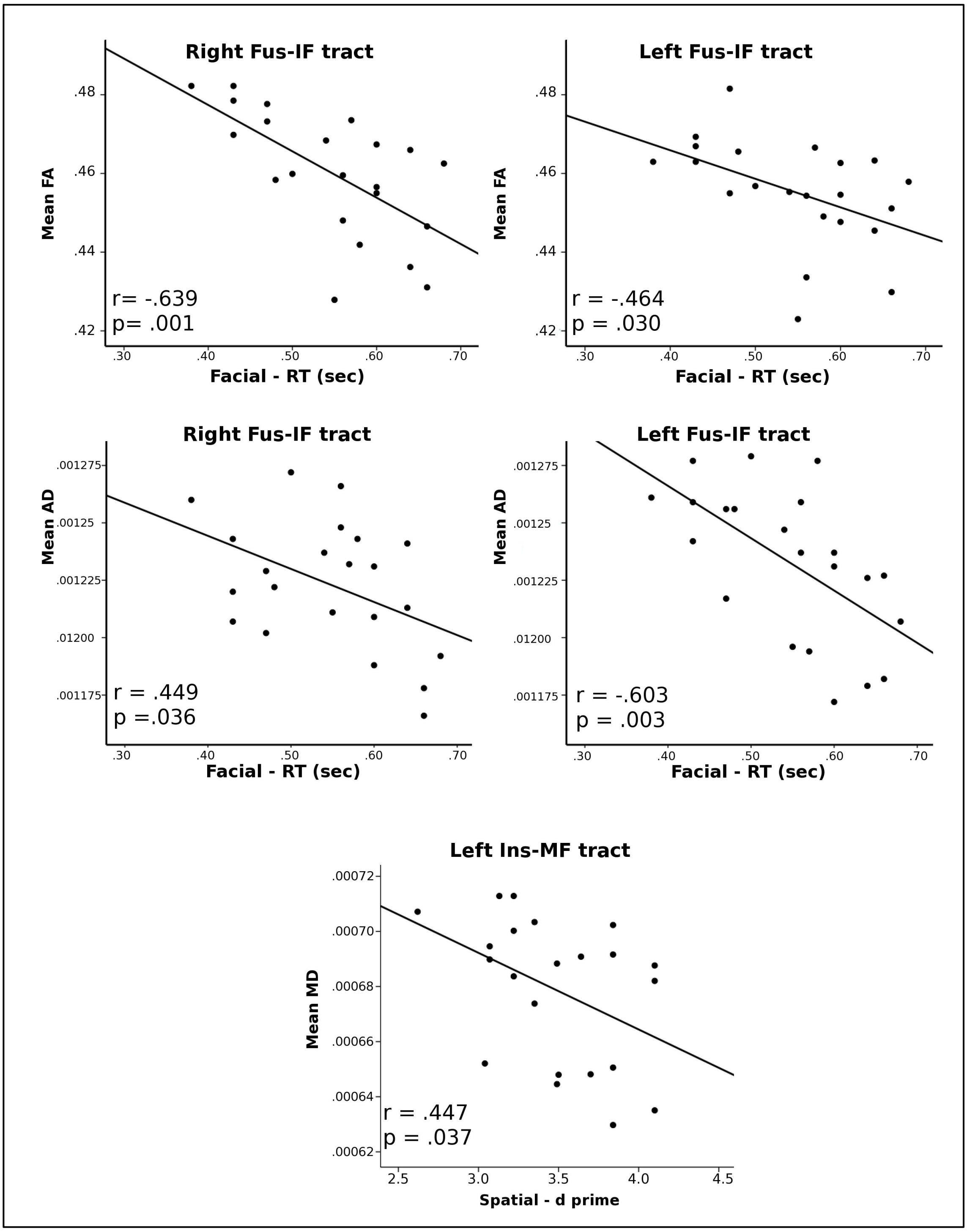

Supplementary Figure 2: Scatter plots of the results showing significant correlations between DTI measures and behavioral results (reported in section 3.5 of the main manuscript).

Fus: Fusiform; *IF*: Inferior frontal; *Ins*: Insula; *MF*: Middle Frontal; *RT*: Reaction time, *FA*: Fractional Anisotropy; *AD*: Axial Diffusivity, *MD*: Mean Diffusivity.
